# Cruciform DNA Structures Act as Legible Templates for Accelerating Homologous Recombination in Transgenic Animals

**DOI:** 10.3390/ijms23073973

**Published:** 2022-04-02

**Authors:** Huan Ou-Yang, Shiao-Hsuan Yang, Wei Chen, Shang-Hsun Yang, Abdulkadir Cidem, Li-Ying Sung, Chuan-Mu Chen

**Affiliations:** 1 Program in Translational Medicine, Department of Life Sciences, National Chung Hsing University, Taichung 402, Taiwan; huan4096052151@alumni.nchu.edu.tw (H.O.-Y.); 178241@cch.org.tw (S.-H.Y.); cidema.kadir@gmail.com (A.C.); 2 Institute of Biotechnology, College of Bioresources and Agriculture, National Taiwan University, Taipei 106, Taiwan; 3 Reproductive Medicine Center, Department of Gynecology, Changhua Christian Hospital, Changhua 515, Taiwan; 4 Division of Pulmonary and Critical Care Medicine, Chia-Yi Christian Hospital, Chiayi 600, Taiwan; peteralfa2004@gmail.com; 5 Department of Physiology, National Cheng Kung University, Tainan 701, Taiwan; syang@mail.ncku.edu.tw; 6 Institute of Basic Medical Sciences, College of Medicine, National Cheng Kung University, Tainan 701, Taiwan; 7 Department of Molecular Biology and Genetics, Erzurum Technical University, Erzurum 25250, Turkey; 8 The iEGG and Animal Biotechnology Center, National Chung Hsing University, Taichung 402, Taiwan; 9 Rong-Hsing Translational Medicine Research Center, Taichung Veterans General Hospital, Taichung 407, Taiwan

**Keywords:** inverted repeat (IR), cruciform, transgene, double-strand break (DSB), DNA repair, homologous recombination (HR), gene edit, CRISPR/Cas9, knock-in, genome stability

## Abstract

Inverted repeat (IR) DNA sequences compose cruciform structures. Some genetic disorders are the result of genome inversion or translocation by cruciform DNA structures. The present study examined whether exogenous DNA integration into the chromosomes of transgenic animals was related to cruciform DNA structures. Large imperfect cruciform structures were frequently predicted around predestinated transgene integration sites in host genomes of microinjection-based transgenic (Tg) animals (*αLA-LPH* Tg goat, *Akr1A1^eGFP/eGFP^* Tg mouse, and *NFκB-Luc* Tg mouse) or CRISPR/Cas9 gene-editing (GE) animals (*αLA-AP1* GE mouse). Transgene cassettes were imperfectly matched with their predestinated sequences. According to the analyzed data, we proposed a putative model in which the flexible cruciform DNA structures acted as a legible template for DNA integration into linear DNAs or double-strand break (DSB) alleles. To demonstrate this model, artificial inverted repeat knock-in (KI) reporter plasmids were created to analyze the KI rate using the CRISPR/Cas9 system in NIH3T3 cells. Notably, the KI rate of the 5′ homologous arm inverted repeat donor plasmid (5′IR) with the ROSA gRNA group (31.5%) was significantly higher than the knock-in reporter donor plasmid (KIR) with the ROSA gRNA group (21.3%, *p* < 0.05). However, the KI rate of the 3′ inverted terminal repeat/inverted repeat donor plasmid (3′ITRIR) group was not different from the KIR group (23.0% vs. 22.0%). These results demonstrated that the legibility of the sequence with the cruciform DNA existing in the transgene promoted homologous recombination (HR) with a higher KI rate. Our findings suggest that flexible cruciform DNAs folded by IR sequences improve the legibility and accelerate DNA 3′-overhang integration into the host genome via homologous recombination machinery.

## 1. Introduction

Directed repeats (DRs) and inverted repeats (IRs) create genome instability and have many functions via the formation of specific secondary structures in the genome [1]. The genome instability of IRs causes cruciform extrusion [2,3]. IRs are partially mutated or deleted during replication failure or enzyme digestion in vivo [4]. Some environmental factors affect DNA conformation, such as salt conditions, epigenetic modification, or DNA-binding protein interactions. For example, 14-3-3 cruciform-binding protein binds to cruciform DNAs and regulates eukaryotic DNA replication [5]. The nucleotide composition of the IR structure is approximately 150 to 200 bp. However, the existence of mismatches and spacers in the IRs’ structures improve the stability of IRs [6].

The instability of the DNA structure due to directed or inverted repeat sequences is the primary cause of genetic disorders, such as Huntington’s disease or type A hemophilia. Type A hemophilia occurs because of clotting FVIII deficiency. Approximately 50% of FVIII-deficient patients have large fragment inversions at intron 1 (int1) and intron 22 (int22) in their *FVIII* gene (*F8*). Notably, a large IR region was found at int22 homolog 2 (int22 h2) and int22 h3 [7]. Although int1 also contains a hotspot for *F8* inversion, only one int1 h region was found. The int1 inversion rate was much lower than the int22 inversion rate [8,9]. There are many inversion hotspots, including the inverted repeat region in autosomal chromosomes, such as hVIPR2 [10] and human chromosome 15q11-q13 [11]. These reports suggest that non-allelic homologous recombination (HR) generally occurs at large inverted repeats. Therefore, we investigated whether cruciform DNA was easier to identify as a legible template for accelerating HR processing.

Current studies revealed that most virus genomes integrate into host chromosomes using inverted repeat sequence machinery, such as inverted tandem repeats (ITRs) from adeno-associated virus (AAV) families [12] and long terminal repeats (LTR) from retrovirus and lentivirus families [13]. Notably, the piggyback transposon system, which was isolated from *Cabbage looper*, contains two terminal repeats [14]. All of the integration mechanisms require specific DNA-binding proteins to import viral DNA into nuclei and recombinant viral DNA into the host genome. However, the AAV vector integrates at double-strand break (DSB) sites without DNA-binding proteins [12,15]. The AAV2 ITR mimics the DSB site to gather DNA repair enzymes [16]. These reports showed that IR sequences acted as motifs of DNA-binding proteins from viruses and interacted with the repair mechanisms of host cells. Taken together, DNA structures or specific recombinases participate in exogenous DNA integration.

Except for viral systems, other transgenic methods in animal fields showed a low efficiency of exogenous DNA integration or required a long time and a high cost to select successful recombinant cells or individuals [17]. Therefore, the discovery of new methods to improve the transgenic efficiency in animal genetic engineering is an important issue. However, the exogenous DNA integration mechanisms were not clearly elucidated using pronuclear microinjection methods with linear DNA or gene editing with CRISPR/Cas9 tools. Newly developed gene-editing tools provide more powerful methods to create site-specific gene knockout (KO) or knock-in (KI) animals. However, the KI efficiency for the large exogenous fragments of gene insertions remains low in all platforms. Single-strand DNA (ssDNA) is the most popular material to increase the KI rate for the generation of gene-edited animals [18,19,20,21]. Previous studies demonstrated that the legibility of ssDNA is better than double-stranded DNA (dsDNA) in DNA repair mechanisms.

The present study hypothesized that the stability of dsDNA with cruciform structures was a factor in template alleles for DNA repair mechanisms. Four transgenic (Tg) or gene-edited (GE) animals were used to study the structural characteristics of the surrounding sequences of integration sites before and after transgene insertion or KI using next-generation sequencing (NGS) and the bioinformatics tool of the UNAFold Web Server “DNA folding form”. We constructed different artificial IR structures in the 5′- or 3′-end of transgenes to elucidate the mechanism of template allele determination that accelerates the KI rate in HR processing.

## 2. Results

### 2.1. Cruciform DNA Structures Are a Hotspot of HR in Transgenic Animal Genomes

**Figure 1:**
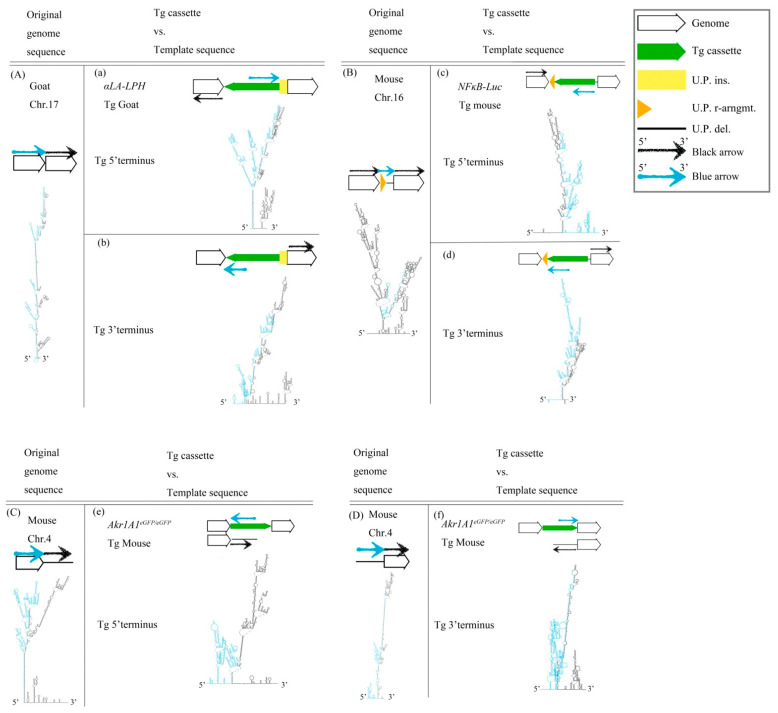
DNA structure of the Tg animal genome before and after Tg integration. (**A**) Goat chr.17 locus, (**B**) Mouse chr.16 locus, (**C**) 5′ terminus, and (**D**) 3′ terminus of mouse chr.4. (**a**–**f**) Descriptions of predicted secondary structures of fusion sequences from the Tg cassette and the predetermined template allele. These secondary structures show the situation of the two sequence assemblies. All sequences are provided in Table S1. Tg cassette, transgene cassette; U.P. ins., unpredictable insertion; U.P. del., unpredictable deletion; U.P. r-arngmt., unpredictable rearrangement. Blue arrow, blue sequences in secondary structures. Black arrow, black sequences in secondary structures.

We were interested in whether some structures surround predestinated transgene (Tg) integration sites of the three Tg animals. The Tg cassettes randomly inserted into the host genomes because those Tg animals were generated by pronuclear microinjection with linear DNA. The host genome sequences were analyzed using NGS and secondary structures were evaluated by the UNAFold Web Server to identify the repair mechanisms used for exogenous DNA integration. Notably, imperfect, large, stem-loop structures were found at approximately 2000 nucleotides (nts) around predestinated Tg integration sites from host genome sequences (Figure 1A–D). The results showed that these regions were hotspots of HR because cruciform structures were present upstream and/or downstream of the target site to create DNA instability. According to the results, we hypothesized that the Tg cassettes integrated into the host genomes via HR. Moreover, the linear Tg cassettes should be 5′- to 3′-resected by the Mre11-Rad50-Nbs1 complex (MRN complex). The linear Tg cassettes should match the predestinated template sequences in the host genomes. Therefore, we fused the sequences of the transgene (Tg) cassette 3′-overhang and the predestinated matched template sequence to predict the secondary structures for finding the match regions. Imperfect stem-loop structures were formed between the Tg cassette 3′-overhang and the predestinated matched template sequence. The data suggested that Tg cassettes imperfectly matched the template DNAs in three different analyzed transgenic animals, including *αLA-LPH* Tg goat (Figure 1a,b), *NFκB-Luc* Tg mouse (Figure 1c,d), and *Akr1A1^eGFP/eGFP^* Tg mouse (Figure 1e,f). The following three observation rules were shown: First, the integration sites occurred in the loop region of cruciform structures. Second, the intervals of predestinated matched templates were replaced. Third, the Tg cassettes remained complete after integration. DNA integration may be highly tolerant of large cis-stem-loop structures using the HR mechanism.

### 2.2. Stability of the Secondary Structure May Relate with Sequence Modification after the HR Mechanism in the Gene-Edited Mouse Genome and αLA-LPH Tg Goat

**Figure 2:**
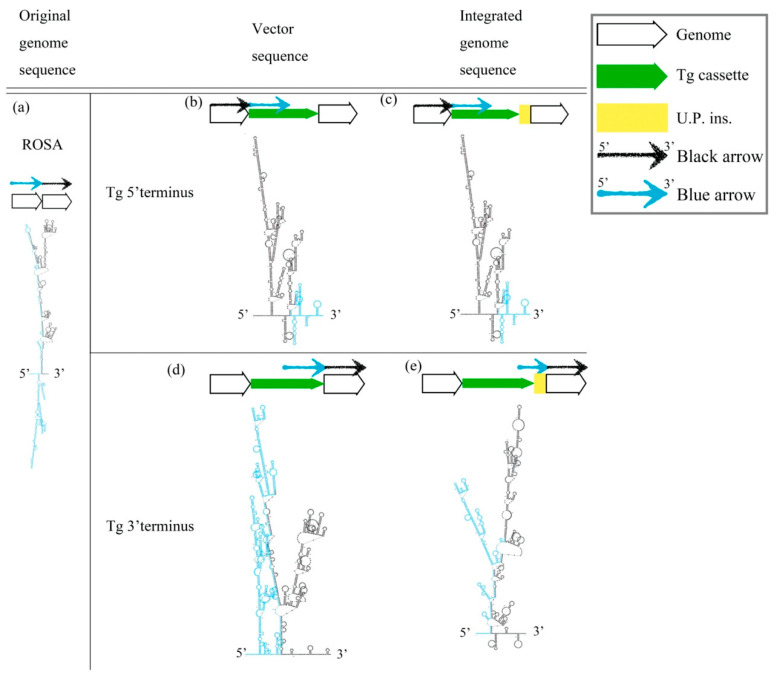
Prediction of the secondary structure of knock-in Tg in the ROSA locus. (**a**) The secondary structure of the mouse ROSA26 locus. (**b**) The 5′ terminal secondary structures of the pDonor-*αLA-Ap1x6*-mROSA plasmid and (**d**) the 3′ terminal secondary structures. Secondary structures of the genome sequence without U.P. ins. (**c**) and with U.P. ins. (**e**) from ICR-*Gt(ROSA)26Sor^em(αLA-Ap1x6)BM2/M^*. All sequences are provided in Table S1. Tg cassette, transgene cassette; U.P. ins., unpredictable insertion. Blue arrow, blue sequences in secondary structures. Black arrow, black sequences in secondary structures.

**Figure 3:**
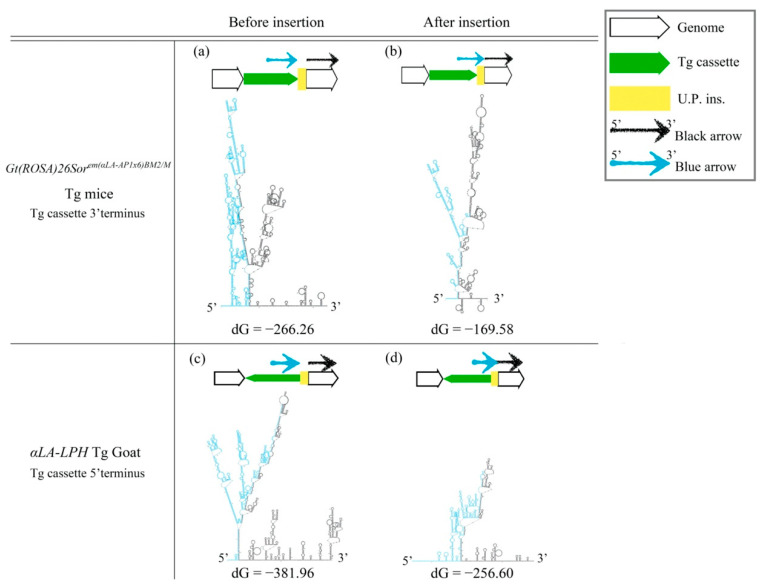
DsDNA stability improved after insertion. (**a**,**c**) Predictions of secondary structures and dG values of the mouse ROSA26 locus and *αLA-LPH* goat Tg locus without the insertion. (**b**,**d**) Predictions of secondary structures and dG values of the mouse ROSA26 locus and *αLA-LPH* goat Tg locus with the insertion. All sequences are provided in Table S1. Tg cassette, transgene cassette; U.P. Ins., unpredictable insertion. Blue arrow, blue sequences in secondary structures. Black arrow, black sequences in secondary structures. dG, Gibbs free energy.

The cruciform DNA structure frequently presented near the predestinated Tg integration sites of transgenic animals. Therefore, we further determined whether the gene-edited animals generated using knock-in methods also showed the same pattern as the Tg animals that were generated via pronuclear microinjection (Figure 2). We analyzed the secondary structure of gene knock-in surrounding sequences in the mouse ROSA locus. A strong stem-loop secondary structure was identified in the ROSA locus (Figure 2a). The ICR-*Gt(ROSA)26Sor^em(αLa-AP1x6)BM2/M^* GE mouse was prepared using the clustered, regularly interspaced, short palindromic repeats/CRISPR-associated protein 9 (CRISPR/Cas9) system to induce a DSB and knock-in the *αLA-AP1x6* Tg cassette in the ROSA26 locus following the HR mechanism. The repair result should be the most precise in our donor Tg cassette. However, a large unpredictable insertion (U.P. ins.) DNA sequence was observed between the Tg cassette 3′-terminus and the ROSA 3′-homologuse arm (HA) (Figure 2d,e). Notably, the Gibbs free energy (dG) value of the stem-loop structure of the integrated genome sequence was higher than the vector sequence. The same situation was observed between the *αLA-LPH* Tg goats with or without the unpredictable insertion (Figure 3). These data suggested that dsDNA is more stable with U.P. ins. than before the insertion. This finding suggests that other repair mechanisms stabilize junction sites by modifying sequences after HR processing. The *αLA-LPH* Tg goat full-genome sequencing data showed that repair mechanisms recombined more than one chromosome fragment in the insertion site. These results suggest that the HR mechanism generated the U.P. ins. (Figure S1).

### 2.3. The Cruciform DNA Structure in HA Improves KI Efficiency

**Figure 4:**
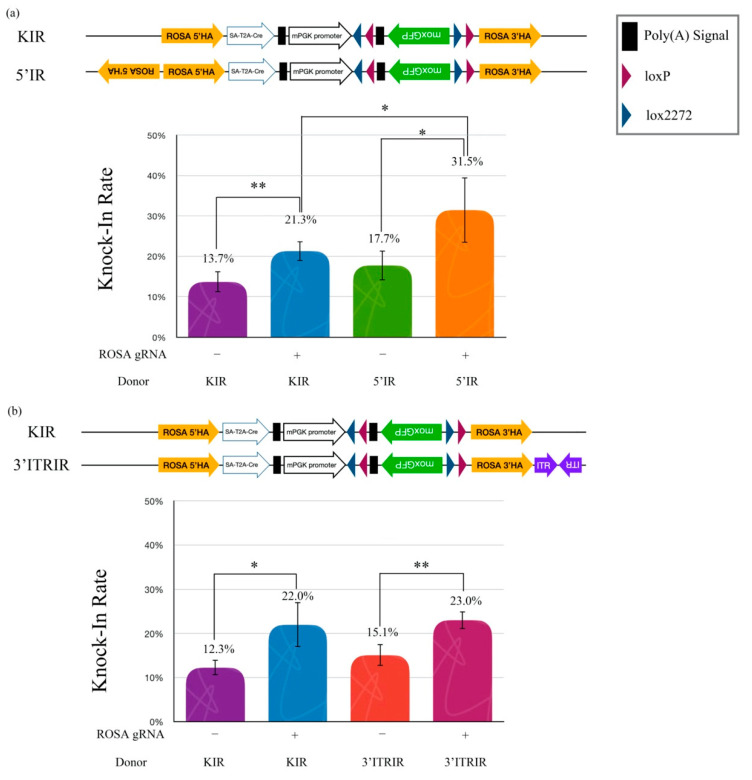
Inverted repeat HA improves the knock-in rate. (**a**) Effects of the knock-in rate by different DNA structures on the HA. (**b**) Effects of the knock-in rate by different DNA structures beyond the HA. Quantitative data of the knock-in rate tests obtained using the flow cytometry. The graph shows the means ± SD of at least two replicates and four individual samples (*n* = 4) for each group. The asterisks (* and **) indicate a significant difference as determined by one-way ANOVA with Fisher’s least significant difference (LSD) test.

We wanted to explain whether the cruciform DNA structure is a legible template for HR. Therefore, three artificial inverted repeat (IR) HA donor plasmids, including a standard knock-in reporter (KIR), a 5′-inverted repeat knock-in reporter (5′IR), and a 3′-extra inverted terminal repeat (ITR)/inverted repeat knock-in reporter (3′ITRIR), were designed to elucidate whether the cruciform DNA actually improved the legibility of HR efficiency in NIH/3T3 cells (Figure 4a). Our hypothesis was that the artificial inverted repeat HA in the donor plasmid would accelerate HR efficiency when a double-strand break (DSB) was created by the CRISPR/Cas9 machinery. The data showed that the KI efficiency of the 5′IR donor with ROSA gRNA (31.5%) was significantly higher than that with the KIR donor (21.3%) (*p* < 0.05). However, the KI efficiency of the 5′IR donor without the ROSA gRNA group was not different from the KIR without the ROSA gRNA group (17.7% vs. 13.7%) (Figure 4a). These results suggest that the 5′ inverted repeat HA improves the HR efficiency in cells. The improvement of KI efficiency only occurred after DSB induction using gene-editing tools.

### 2.4. Only the Presence of the Cruciform Structure in HA Improves the KI Efficiency

We further examined whether the location of the cruciform structure was an important issue in KI efficiency. An inverted repeat AAV2-ITR sequence (ITRIR) was constructed outside of the ROSA 3′HA. After transfection, the KI efficiency of ROSA 3′HA (3′ITRIR) did not show improvement compared to the KIR with ROSA gRNA group (23.0% vs. 22.0%) (*p* = 0.318) (Figure 4b). Our data suggested that the KI efficiency of the HR mechanism was only improved by the cruciform structure located in the HA sequence.

## 3. Discussion

This study provides three main findings: (a) We observed an inverted repeat structure that was frequently present at Tg integration sites and genome inversion hotspots. (b) We are the first group to hypothesize a putative model of the transgene integration mechanism via HR processing and use the gene-editing tool to analyze the effects of cruciform DNA structures in the HR mechanism. (c) We successfully demonstrated that the presence of an extra inverted repeat structure in the HA region of the 5′IR plasmid construct significantly increased the KI efficiency under gRNA guidance, but not in the 3′ITRIR plasmid construct that contained an inverted ITR repeat outside of the HA region.

**Figure 5:**
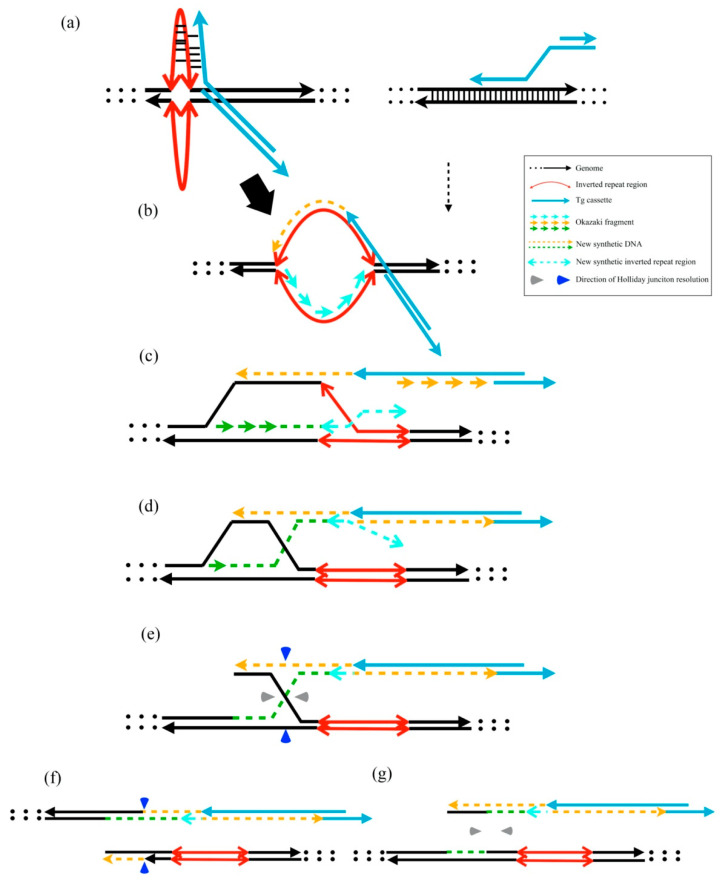
Putative model of transgene cassette integration via the HR mechanism. (**a**) Representative cruciform extrusions from inverted repeat regions and normal HR template without cruciform structures. The 3′-overhang of linear DNA integration into the extruded cruciform DNA is easier than the dsDNA after 5′ to 3′ resection by the MRN complex. (**b**,**c**) Initiation of new DNA synthesis and D-loop migration. (**d**) The lagging strand 3′ terminus assembles the leading strand. (**e**) Holliday junction formation after DNA synthesis. (**f**) When the Holliday junction is resolved through the vertical axis, the Tg cassette joins the gDNA. (**g**) When the Holliday junction is resolved through the horizontal axis, the Tg cassette does not join the gDNA.

According to our findings, we proposed a putative model for exogenous DNA insertion cross-reacting via the stem-loop structure of host genomes. The instability of the template DNA with several inverted repeat cruciform DNA structures improves recombination enzyme recognition. The template DNA was easily integrated via the 3′-overhang of linear exogenous DNAs (Figure 5a–c). The linear DNAs were linked with gDNA via asymmetric Holliday junction resolution (Figure 5d–g). The HR or other repair mechanisms may insert unpredicted sequences into junctions of Tg cassettes and host genomes [22]. The addition of dG values after unpredicted insertion may provide the evidence that the gDNA stability was related to the unpredicted modification.

Several mechanisms may explain the improvement in template DNA legibility by inverted repeat sequences. First, inverted repeat sequences are relatively unstable in vivo [23,24]. Therefore, HR enzymes, especially Rad51 and Rad54, may easily identify inverted repeat sequences. Second, inverted repeat sequences are likely the origin of DNA replication [5,25]. The DNA replication mechanism may help the template DNA amplify itself and maintain the half-life in cells. HR enzymes are more activated during DNA replication in S phase [26]. Therefore, HR enzymes are more likely to recognize the template DNA. Third, many types of cruciform DNA-binding proteins (CBPs), such as Rad51AP1 [27,28], Rad54 [29], 14-3-3 [30], PARP-1 [31], p53 [32], and BRCA1 [33], play roles in DNA replication and repair in mammalian cells [34]. These reports suggested that the structural conformation of inverted repeat DNA strongly interacted with mechanisms of DNA replication and repair. Our data provided two new pieces of evidence. First, gDNA sequences around the predestinated Tg integration site generally formed stem-loop structures. Second, the artificial cruciform DNA structure played a role in improving HR for the template DNA in vivo. Therefore, the cruciform DNA structure increased the DNA legibility in vivo via the HR mechanism.

AAV viruses are useful tools for long-term maintenance in mammalian cells without integration into host genomes. The recombinant AAV (rAAV) system is generally episomal concatemerization (>99.99%) in vivo [35], which includes cruciform DNA structures at ITR-IR regions of episomes. The cruciform structure was formed via HR after second-strand DNA synthesis. The episomal concatemer is highly persistent in nuclei in a chromatin-like structure [36]. AAV-ITR-IR may play a key role in increasing plasmid persistence in vivo [37]. The AAV ITR sequence includes transcriptional activity in vivo [38]. Notably, the transcriptional silencing of rAAV in mammalian cells is related to the interaction between the ATM/MRN complex and T-shaped ITRs [39,40,41]. These reports indicate that the ITR structure is the key that mediates rAAV interaction with host cells. Notably, some inverted repeat sequences, such as AAV2 ITR, AAVS1, and Alu long inverted repeats [42], slightly induce the HR repair mechanism [16]. This previous study suggested that cruciform DNA structures induced HR and directly replaced the structure by inducing a DSB. Our study also showed that all four transgene cassettes integrated into cruciform DNA structures of insertion sites in Tg animals. However, we found that predestinated matched templates, which imperfectly matched with Tg cassettes, were near integration sites in host chromosomes without DSB. All predestinated matched templates were replaced by Tg cassettes. Therefore, assembly regions were not found after DSB. Our data suggested that Tg cassettes integrated and imperfectly matched with assembly regions before DSB. Therefore, cruciform DNA structures act as templates for Tg integration in the HR mechanism, but cruciform DNA structures did not induce DSB before Tg cassette integration.

Platforms for large IR plasmid construction and amplification are not stable. Therefore, it is a critical to improve methods of IR plasmid production to deeply analyze the relationship between DNA structures and the HR mechanism. Some commercially competent cells are compatible with plasmids, including unstable structures, such as Stbl strains (Invitrogen), SURE strains (Agilent), Endura strains (Lucigen), and NEB stable strains (New England Biolabs). Although commercially competent cells may be used, the stability and yield of plasmid production is dependent on plasmids. The plasmid yield of 5′IR in the SURE2 strain was lower than in the DH5α strain (ECOS101^TM^) in our study (data not shown). The closed linear plasmid from Lucigen may overcome issues of unstable DNA construction and amplification. Although these companies use their products to overcome unstable plasmid production, individual customers should test the precise protocol.

Gene-editing methods were developed over the past decade. KO and KI induce DNA double-strand breaks using protein- or RNA-guided site-specific endonucleases, such as zinc-finger nucleases (ZFNs) [43], transcription activator-like effector nucleases (TALENs) [44], and CRISPR/Cas9 [45]. After DSB, cells repair their DNA via non-homologous end-joining (NHEJ) or HR [46]. Most NHEJ repairs result in insertion–deletion mutations. Genes generally lose functions when NHEJ repair sites occur at the coding DNA sequence (CDS), which is called the gene KO. The HR mechanism uses multiple steps to repair DNA. The donor DNA integrates into the DSB locus when the donor DNA is used as the HA template. However, the HR efficiency is much lower than NHEJ in cells [47,48], which explains the lower KI rate compared to the KO rate [49]. Therefore, understanding the NHEJ/HR switch mechanism is the foundation for improving HR efficiency. Table 1 summarizes the factors involved in the NHEJ/HR switch mechanism, including repair enzymes and DNA conformation. For repair enzyme factors, the activities of DSB repair enzymes were manipulated to improve the KI efficiency in recent reports, such as HR enzyme overexpression or RS-1 addition [50,51,52] for approximately 0–20%, NHEJ inhibitor addition [53,54,55] for approximately 0–40%, and small molecule addition to arrest the cell cycle [56,57] for approximately 20–40% of KI efficiency improvements. However, these reports showed a wide range of results and were not consistent in KI improvement.

For the DNA conformation factors, the distance between the template and the host DNA was manipulated to improve KI efficiency using a modified biotin–streptavidin approach to localize repair templates to target sites. This study hypothesized that the distance of two alleles would significantly affect HR efficiency [58]. However, their results showed a high variation in KI efficiency. Current experimental data suggest that the variation in KI efficiency is largely sequence-dependent [59]. However, these data provide no clear answers of how DNA sequences affect KI efficiency. Moreover, we were interested in which is sequence-dependent in the HR mechanism, either DSB alleles or template alleles. Notably, the KI efficiency of the 5′IR plasmid was better than the KIR plasmid in our report. These data suggested that the template DNA containing the unstable cruciform DNA structure improved the HR efficiency. Therefore, the structure/stability-dependency may be more important in the template allele than in the DSB allele.

**Table 1:** Factors of the NHEJ/HR switch mechanism.

Factors	Summary	References
Repair Enzymes		
Activation of HR pathway	Weak improvement of KI efficiency by HR enzyme overexpression or RS-1 addition (0~20%)	[50,51,52]
Inhibition of NHEJ pathway	Moderate improvement of KI efficiency by NHEJ inhibitors (0~40%)	[53,54,55]
Arrest of cell cycle	Strong improvement of KI efficiency by using small molecules to arrest cell cycle (20~40%)	[56,57]
DNA conformation		
The distance between the template DNA and the host DNA	Strong improvement of KI efficiency by modifying donor DNA (20~40%)	[58]
The structure of the template DNA	Indirect evidence showed that the cruciform structure from ITR affected KI efficiency	[60,61]
	Our data suggested that the cruciform structure improved KI efficiency (10~20%)	This study
The transcription activity of the DSB allele	High level of transcription activity around DSB sites induced HR via Rad52 activation and 53BP1 inhibition	[62,63,64]
	DNA:RNA hybrid forms are related to DNA repair mechanisms at DSB loci	[65,66]

## 4. Materials and Methods

### 4.1. Transgenic Animal Production

Four transgenic animals were created using pronuclear microinjection (PN inj.) in our laboratory. Bovine alpha-lactalbumin promoter-driven lactase-phlorizin hydrolase (*αLA-LPH*) transgenic (Tg) goats, *Akr1A1* gene knockout with *CMV-eGFP* knock-in (*Akr1A1^eGFP/eGFP^*) Tg mice [67], and NFκB promoter-driven luciferase (*NFκB-Luc*) Tg mice [68] were created via the traditional PN inj. using linearized DNAs. The ICR-*Gt(ROSA)26Sor^em(αLa-AP1x6)BM2/M^* Tg mice were created via PN inj. using the CRISPR/Cas9 gene-editing tool and circular donor DNA. All animal experimental procedure was approved by the Institutional Animal Care and Use Committee of National Chung Hsing University (code: IACUC No. 108-029; date: 1 July 2019).

### 4.2. Analysis of Transgenic Cassettes in the Integration Sites of Chromosomes

#### 4.2.1. *αLA-LPH* Tg Goats

The whole-genome sequencing (WGS) method was used to analyze the Tg integration site in the *αLA-LPH* Tg goat genome. Genomic DNA was isolated from the blood of α*LA-LPH* Tg goats using a QIAamp^®^ DNA Blood Midi kit (51185; QIAGEN Sciences Inc., Germantown, MD, USA). The sequencing library was generated using a Truseq Nano DNA Library Prep Kit (#20010965; Illumina Inc., San Diego, CA, USA) following the manufacturer’s recommendations, and index codes were added to each sample. Briefly, the genomic DNA sample was fragmented by sonication to approximately 350 bp. The DNA fragments were end-polished, A-tailed, and ligated with the full-length adapter for Illumina sequencing, followed by further PCR amplification. PCR products were purified in an AMPure XP system (A63881; Beckman Coulter Life Science, Indianapolis, IN, USA), and libraries were analyzed for size distribution using an Agilent bioanalyzer with an Agilent High-Sensitivity DNA kit (#5067-4626; Agilent Technologies Inc., Santa Clara, CA, USA) and quantified using a Qubit fluorometer with dsDNA HS assay kit reagent (Q32854; Thermo Fisher Scientific Inc., Waltham, MA, USA). The DNA libraries were sequenced on an Illumina NovaSeq 6000 platform (Illumina Inc., San Diego, CA, USA), and 150 bp paired-end reads were generated by Genomics BioSci & Tech Co. (Taipei, Taiwan).

#### 4.2.2. *Akr1a1^eGFP/eGFP^* Tg Mice

Our previous report used the compatible ends ligation inverse-PCR (CELI-PCR) method to analyze the *Akr1a1^eGFP/eGFP^* Tg integration site. Briefly, genomic DNA was isolated from the tail snip of *Akr1a1^eGFP/eGFP^* Tg mice. The genomic DNA was digested with *Bgl*II and *Bam*HI (NEB, Ipswich, MA, USA). Digested DNA fragments were ligated into a circular form. The targeted DNA was amplified using inverse PCR from circular DNA. PCR products were cloned into the pGEM-T Easy vector (A1360; Promega, Madison, WI, USA). Plasmid DNAs from a single colony were isolated and used for Sanger sequencing.

#### 4.2.3. *NFκB-Luc* Tg Mice

The targeted locus amplification–next-generation sequencing (TLA-NGS) method was used to analyze the genome of *NFκB-Luc* Tg mice in this study. We isolated primary tail-snip fibroblasts from *NFκB-Luc* Tg mice. The cells were fixed with 2% formaldehyde, and the genomic DNA (gDNA) was subjected to in-cell digestion by *Hin*1II-FD (FD1834; Thermo Fisher Scientific Inc., Waltham, MA, USA). The digested gDNAs were further ligated using T4 ligase (15224041; Thermo Fisher Scientific Inc.). After re-crosslinking formaldehyde using the phenol/chloroform and ethanol precipitation method, DNAs were digested by *Xce*I-FD (FD1474; Thermo Fisher Scientific Inc.). The TLA template was prepared via a second ligation using T4 ligase (15224041; Thermo Fisher Scientific Inc.). We used the primer sets TLA-L and TLA-R to amplify the target region using PfuUltra II Fusion HS DNA polymerase (600672; Agilent Technologies Inc., Santa Clara, CA, USA). The Celero PCR Workflow with enzymatic fragmentation library preparation Kit (9363; TECAN, Seestrasse, Männedorf, Switzerland) was used to construct the DNA library. The NGS library was analyzed via paired-end sequencing with a MiSeq system (SY-410-1003; Illumina Inc., San Diego, CA, USA) by Tri-I Biotech Inc. (Taipei, Taiwan).

#### 4.2.4. *Gt(ROSA)26Sor^em(αLA-AP1x6)BM2/M^* Tg Mice

The transgene junction of ICR-*Gt(ROSA)26Sor^em(aLa-AP1x6)BM2/M^* was amplified using PfuUltra II Fusion HS DNA polymerase (600672; Agilent Technologies Inc.). PCR products were analyzed via Sanger sequencing.

### 4.3. Secondary Structure Prediction

The UNAFold Web Server “DNA folding form” (http://www.unafold.org/mfold/applications/dna-folding-form.php, accessed on 19 August 2021) was used to analyze the secondary structure of approximately 2000 nt of the surrounding sequences in Tg integration sites [69]. We chose “polymer” in correction type, “Untangle with loop fix” in structure draw mode, and “Flat” or “Flat-alt” in exterior loop type for all analyses. Other options were used in the default setting. All sequences were deposited into the National Center for Biotechnology Information (NCBI) database (https://www.ncbi.nlm.nih.gov accessed on 22 October 2021). The analytic intervals are provided in Table S1.

### 4.4. Construction of Knock-In Reporter Plasmids

We established a KI efficiency reporter system using the FLEX system. The SA-T2A-Cre cassette was constructed downstream of ROSA 5′HA in the pDonor MCS Rosa26 (a gift from Charles Gersbach; Addgene plasmid #37200; http://n2t.net/addgene:37200 (accessed on 30 November 2015); RRID: Addgene_37200). The FLEX system was constructed downstream of the SA-T2A-Cre cassette. The KIR donor plasmid included ROSA 5′HA, SA-T2A-Cre, the FLEX system, and ROSA 3′HA. The 5′IR donor plasmid was constructed via the addition of the inverse ROSA 5′HA to the KIR donor plasmid. The 3′ITRIR donor plasmid was constructed by adding an inverted AAV2 ITR sequence to the KIR donor plasmid.

### 4.5. Cell Culture and Transfection

NIH/3T3 cells (CRL-1658; ATCC, Manassas, VA, USA) were cultured in Dulbecco’s modified Eagle’s medium (11965; Thermo Fisher Scientific Inc.) supplemented with 10% fetal bovine serum (26140079; Thermo Fisher Scientific Inc.) at 37 °C in air containing 5% CO_2_. For KI rate measurement, NIH/3T3 cells were seeded in 24-well plates and co-transfected with CRISPR/Cas9 plasmid (pU6-(BbsI) CBh-Cas9-T2A-mCherry or pU6-(sgROSA)_CBh-Cas9-T2A-mCherry) (a gift from Ralf Kuehn; Addgene plasmid #64324; http://n2t.net/addgene:64324 (accessed on 30 November 2015); RRID: Addgene_64324) and donor plasmid using LipofectamineTM 2000 (11668030; Thermo Fisher Scientific Inc.). The sgROSA gRNA target sequence is “GACTCCAGTCTTTCTAGAAGA”.

### 4.6. Quantitation of Knock-In Rates

KI rates were measured 24 h post-transfection using flow cytometry (Accuri^TM^ C6 Plus; BD Bioscience, East Rutherford, NJ, USA) with 20,000 transfected cells per sample. Red fluorescence-positive cells were used as the control for transfection efficiency. Green (G) and red (R) fluorescence-positive cells (G + R) indicated successful transfection and KI. The KI rate was determined by (G + R)/R. The background signals of the knock-in reporter system and transfection efficiencies were tested for finding the optimal condition to ignore the leak expression (Figures S2–S4) [70]. We used an optimal transfection condition of co-transfection of the 50 ng donor plasmid and 1.8 µg CRISPR/Cas9-mCherry all-in-one plasmid per well in 24 wells (Figure S3).

### 4.7. Statistical Analysis

The results are expressed as the means ± SD (standard deviation). Statistical analyses were performed using one-way ANOVA with Fisher’s least significant difference (LSD) test in SPSS software (Version 20, IBM Corp., Armonk, NY, USA). *p* < 0.05 was considered statistically significant (* *p* < 0.05 and ** *p* < 0.01).

## 5. Conclusions

We first reported the presence of cruciform DNA structures of genome sequences in the transgene integration sites analyzed in four different strains of Tg animals. We found that the ROSA26 locus exhibited a similar cruciform DNA structure in the knock-in site. We analyzed the potential function of cruciform DNA structures during HR processing using the CRISPR/Cas9 knock-in system. Notably, our data suggested that the cruciform DNA structure in the HA region improved the knock-in rate. In conclusion, the inverted repeat sequence may be more legible and recognized as a template for the HR mechanism based on the structural instability of the cruciform DNA.

## Data Availability

The data presented in this study are available upon request from the corresponding author.

## Supplementary Materials

The following supporting information can be downloaded at: https://www.mdpi.com/article/10.3390/ijms23073973/s1.
