# Supplementary material for: Cruciform DNA Structures Act as Legible Templates for Accelerating Homologous Recombination in Transgenic Animals
Source: Int J Mol Sci. 2022 Apr 2;23(7):3973. doi: 10.3390/ijms23073973 (PMC9000021; doi:10.3390/ijms23073973)
Supplement: Supplementary file 1 [file ijms-23-03973-s001.zip › ijms-1635322-supplementary.pdf]

Supplementary Data

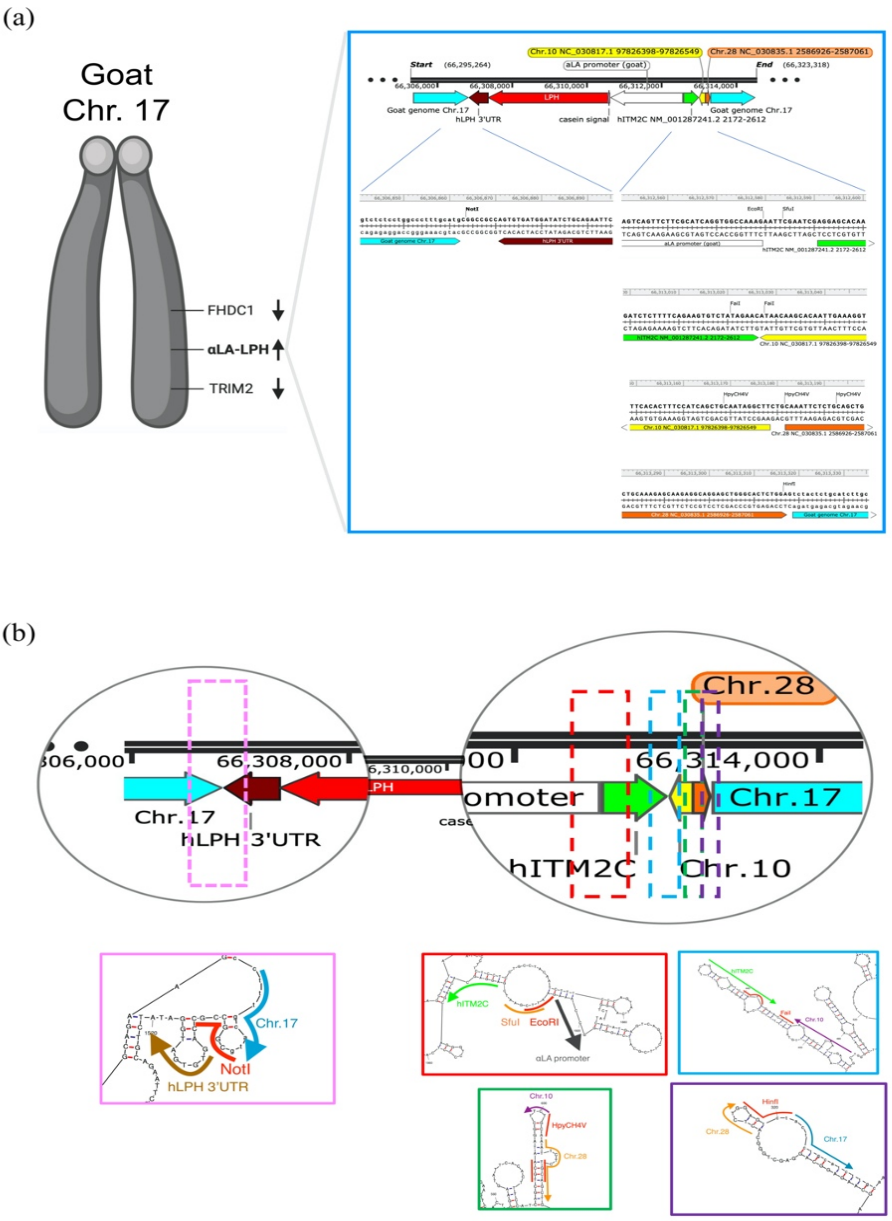

Supplementary Figure S1. Multiple chromosome rearrangement in the *αLA-LPH* Tg goat genome. (**a**) Descriptions of the multiple chromosome rearrangement at the 3’ junction site of goat chromosome 17. (**b**) All junctions included multiple types of unstable structures, such as stem-loop structures and palindromic sequences.

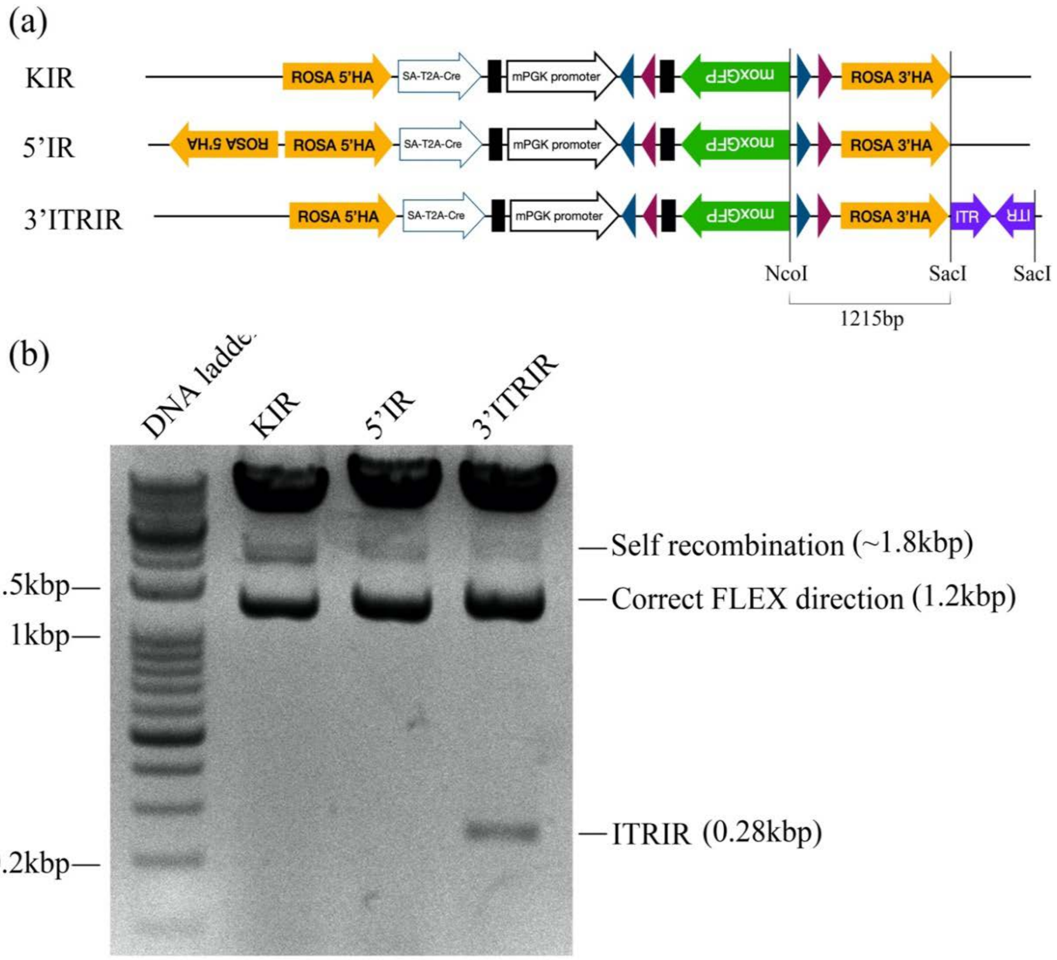

32.7%	26.7%	20.6%	Self Recombination
67.3%	73.3%	79.4%	Correct FLEX direction

Supplementary Figure S2. Quantification of plasmid self-recombination in *E. coli*. (**a**) Description of cutting sites in the three plasmid construction maps. (**b**) The image shows bands of correct FLEX direction (1.2 kbp), self-recombination (~1.8 kbp), and ITRIR (0.28 kbp) after digestion with *Nco*I and *Sac*I followed by 1.5% agarose gel electrophoresis in 0.5x TAE buffer and 20 min at 100 V. The ratios between correct FLEX direction and self-recombination were calculated using ImageJ software.

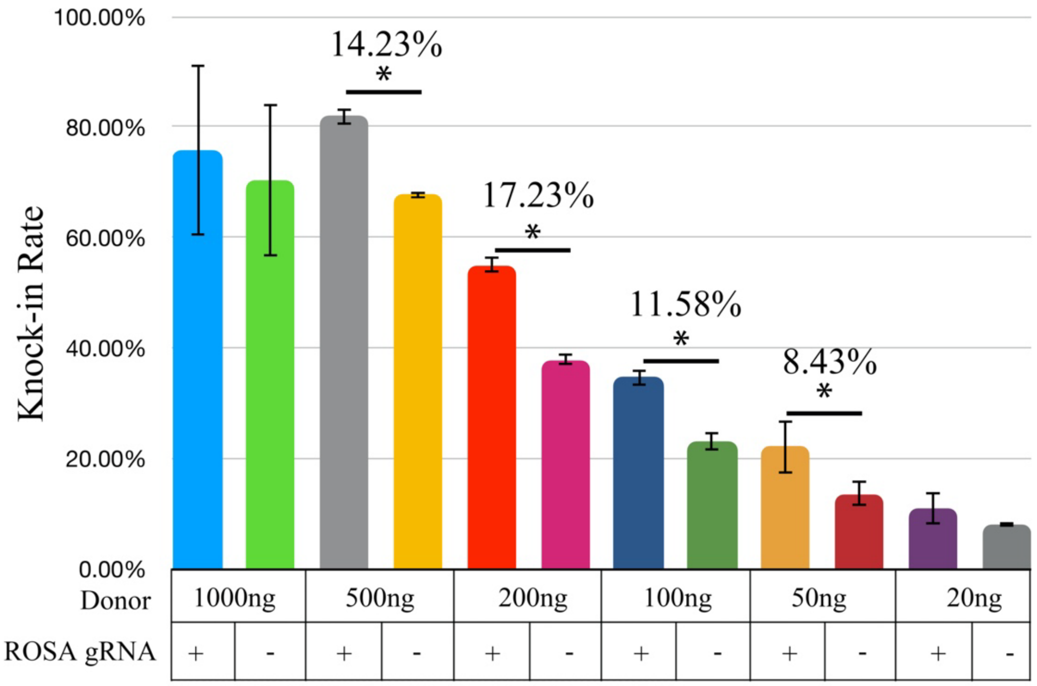

Supplementary Figure S3. Knock-in reporter (KIR) background and donor plasmid dose tests. Quantitative data of the KIR background and the dose tests of donor plasmids obtained using flow cytometry. The graph shows the means ± SD of at least two replicates and n = 3 for each group. Knock-in rates were calculated following the equation of percentages of green- and red-positive cells divided by percentages of total red-positive cells. The asterisk (*) shows significant differences ($P < 0.05$) according to the F test and Student's t test.

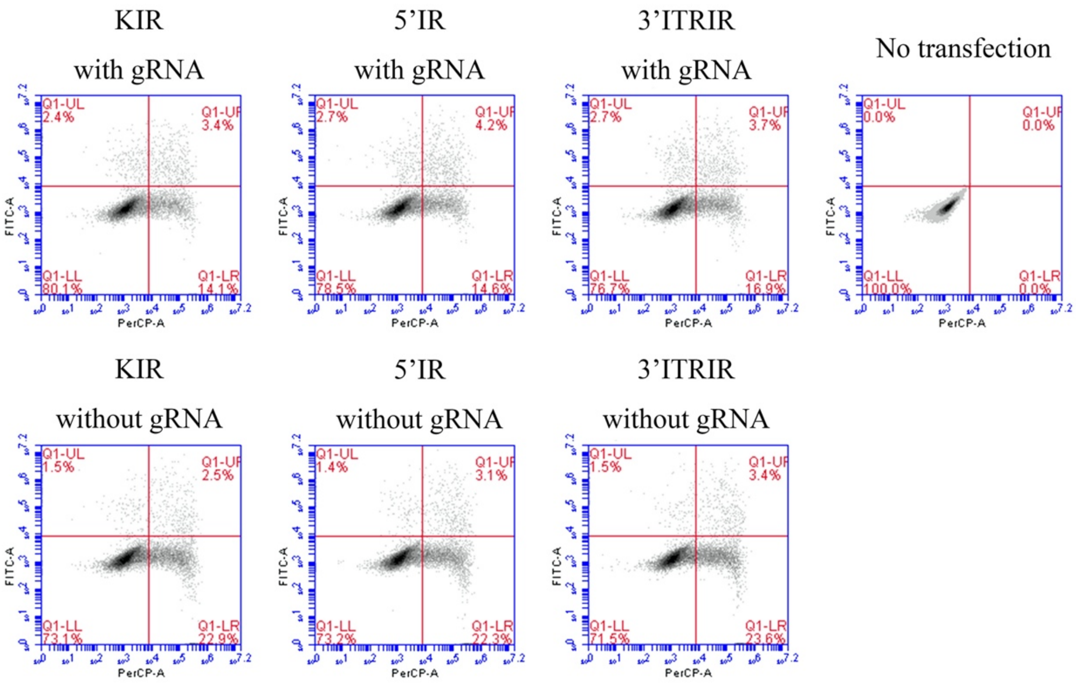

Supplementary Figure S4. Knock-in efficiency data by flow cytometry analysis. A total of 20,000 cells were detected in each condition. The FITC-A and PerCP-A Channels show the signal of the Green and Red positive cells. The Q1-UF region means the Green (G) and Red (R) fluorescence-positive cells. The Q1-UF and Q1-LR regions are the Red (R) fluorescence-positive cells. The KI rate was determined by (G+R)/R.

Supplementary Table S1. The deposited accession numbers in NCBI of the secondary structure prediction sequences in this study

Fig.	NCBI Reference Sequence or Accession Number	Blue arrow	Black arrow	Tg cassette size
1A	NC_030824	66,305,817 - 66,306,862	66,306,863 - 66,307,823	No Tg cassette
1B	NC_000082	50,613,629 - 50,614,619 (Left)<br>50,614,821 - 50,615,628 (Right)	50,614,620 - 50,614,820	No Tg cassette
1C	NC_000070	116,495,816 - 116,496,825	116,496,826 - 116,497,815	No Tg cassette
1D	NC_000070	116,532,279 - 116,533,278	116,533,279 - 116,534,279	No Tg cassette
1a	OK586153	6,436 - 7,435	673 - 1,718 (Reverse complement)	5,709 bp
1b	OK586153	1,719 - 2,679 (Reverse complement)	8,175 - 9,376	
1c	OK586149	2,173 - 3,172 (Reverse complement)	2 - 1,001	2,100 bp
1d	OK586149	3,173 - 4,172	1,062 - 2061 (Reverse complement)	
1e	OK586152	3,120 - 3,972 (Reverse complement)	-	3,252 bp
	NC_000070	-	116,496,826 - 116,497,815	
1f	OK586152	5,222 - 6,372 (Reverse complement)	-	
	NC_000070	-	116,533,279 - 116,534,279	
2a	NC_000072.7	113,052,181 - 113,052,992	113,052,993 - 113,053,786	No Tg cassette
2b	OK586151	658 - 1,452	1,453 - 2,956	2,925 bp
2c	OK586150	1 - 795	796 - 1,300	
2d	OK586151	3,290 - 4,319	4,320 - 5,132	
2e	OK586150	2,634 - 3,720	3,721 - 4,533	
3a	OK586151	3,290 - 4,319	4,320 - 5,132	2,925 bp
3b	OK586150	2,634 - 3,720	3,721 - 4,533	
3c	OK586153	6,436 - 7,435	8,175 - 9,376	5,709 bp
3d	OK586153	7,370 – 8,174	8,175 – 9,376	
